# Transient Intracranial Circulatory Arrest Evidenced at the Time of Intracranial Aneurysm Rupture: Case Report

**DOI:** 10.1007/s12028-020-00948-w

**Published:** 2020-03-26

**Authors:** Ilari Rautalin, Miikka Korja

**Affiliations:** Department of Neurosurgery, University of Helsinki and Helsinki University Hospital, Topeliuksenkatu 5, P.O. Box 266, 00029 Helsinki, Finland

## Introduction

One-fourth of people with aneurysmal subarachnoid hemorrhage (SAH) die from the bleed suddenly in emergency rooms or before reaching hospital [1]. Unless medicolegal or medical autopsies are systematically performed to all sudden deaths, these SAH deaths are almost invariably classified as sudden cardiac deaths. The first observable symptom of sudden SAH death is loss of consciousness (LOC), which is also a common presenting symptom of non-fatal SAH. The pathophysiology of LOC at the time of aneurysm rupture is controversial. However, similar to sudden SAH deaths, the etiology of LOC in non-fatal SAHs is often considered to be cardiogenic. This presumption may, however, stem from the lack of evidence suggesting other etiologies.

This report illustrates that a transient intracranial circulatory arrest associates with LOC and can occur without cardiac arrest. Furthermore, the case shows that patients experiencing a transient intracranial circulatory arrest can recover relatively well, despite the poor clinical status at the time of bleed.

### Clinical Presentation

Reporting follows the CARE guideline [2]. According to the Finnish legislation, the patient consent is not required to use de-identified patient information for scientific purposes.

#### Presenting Symptoms

A 50- to 60-year-old ex-smoker with hypertension and asthma lost consciousness while playing sports. Bystanders performed cardiopulmonary resuscitation, and the patient regained consciousness in a couple of minutes.

#### Clinical Findings

**Fig. 1:**
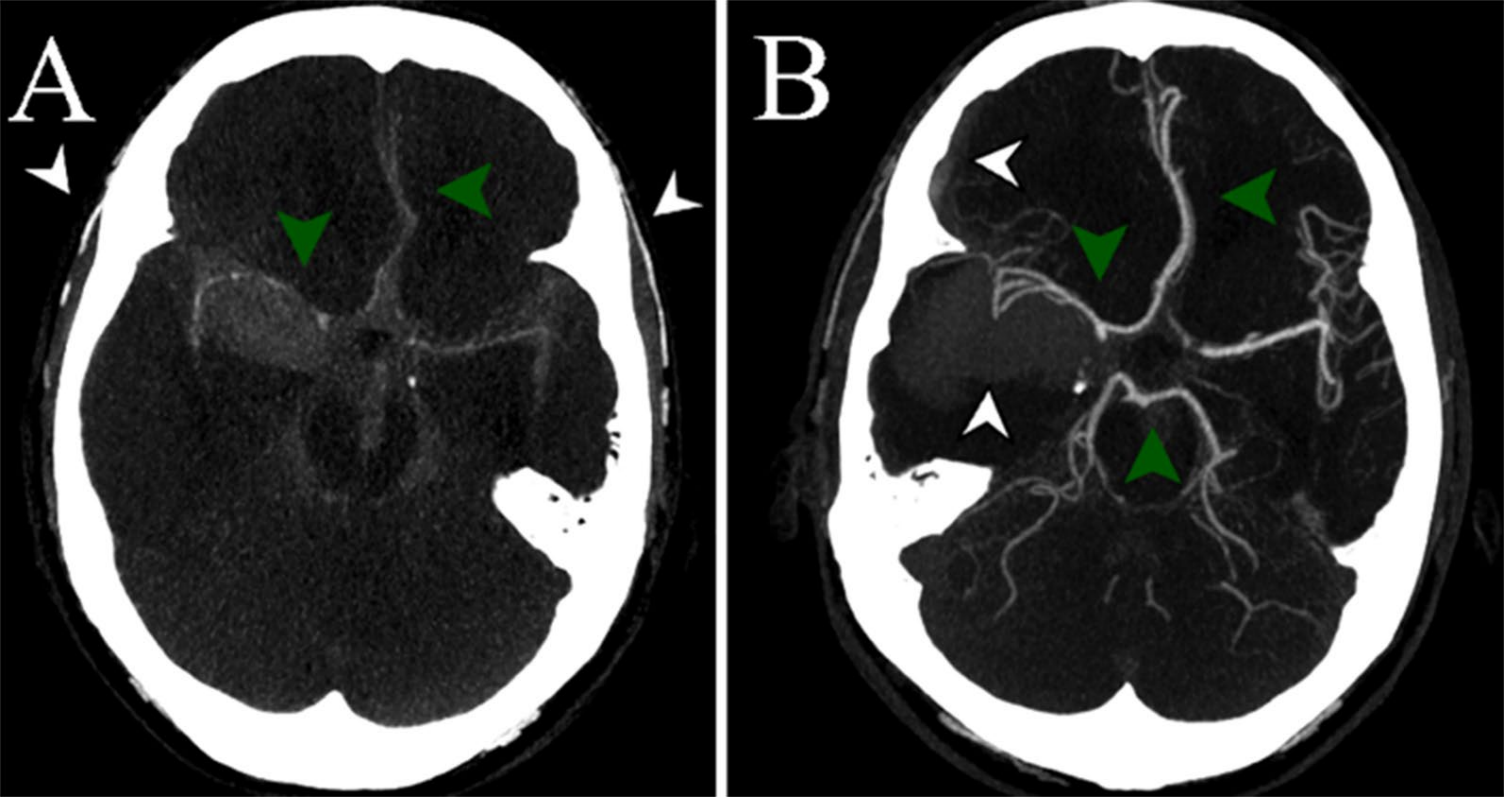
Computed tomography angiography (CTA) scans. At the time of aneurysm rupture and intracranial circulatory arrest (**a**), anterior and middle cerebral arteries filled vaguely (**a**; green arrowheads) whereas extracranial temporal arteries were clearly viewed (**a**; white arrowheads). After regaining intracranial circulation (**b**), anterior, middle and posterior cerebral arteries were filling normally again (**b**; green arrowheads). CTA showed also signs of a rebleed, such as a grown intracerebral hematoma and a new right acute subdural hematoma (**b**; white arrowheads). Computed tomography scan confirmed the rebleed (images not shown) (Color figure online)

**Fig. 2:**
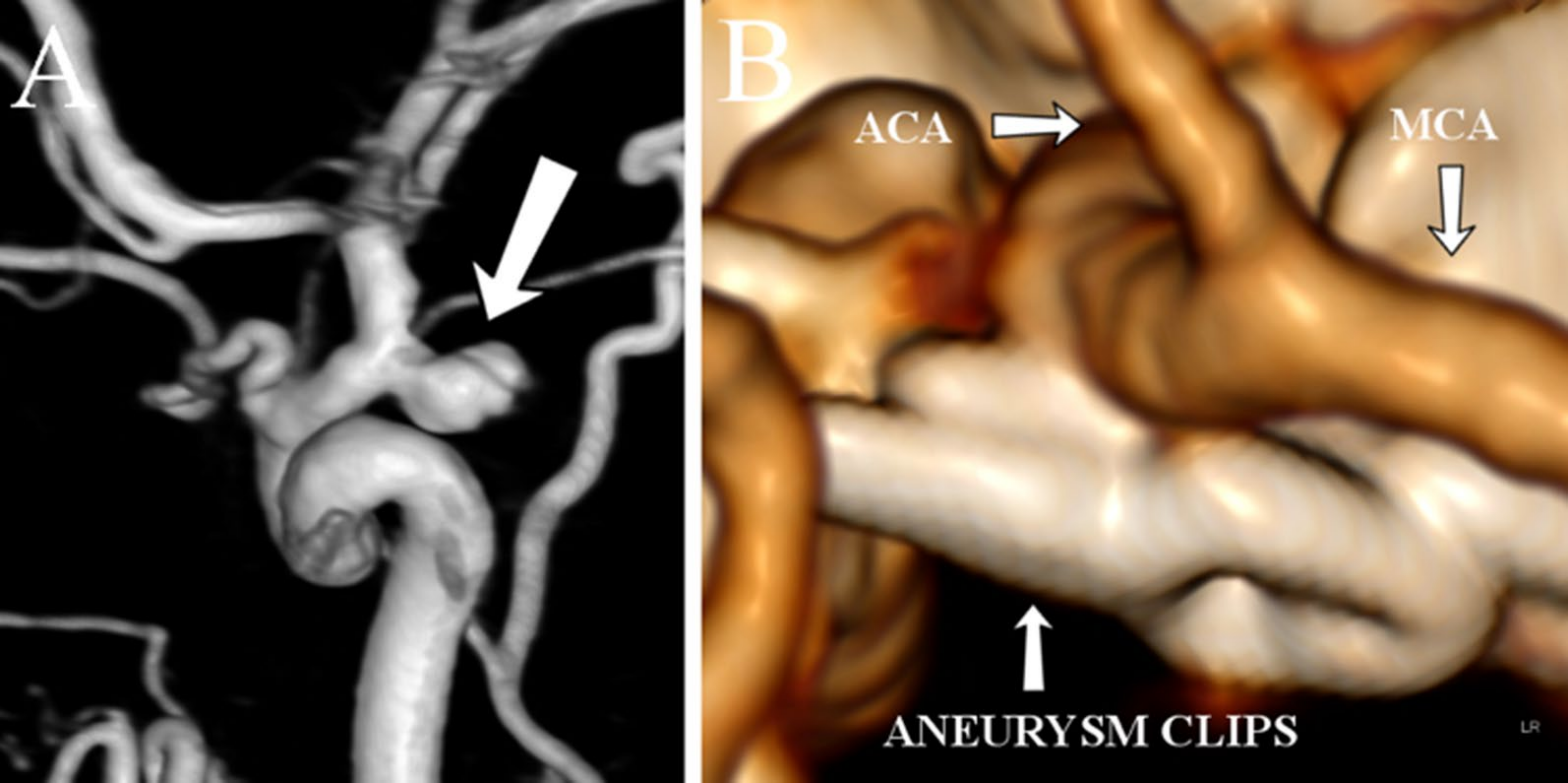
A conventional catheter angiography showed a right posterior communicating artery aneurysm (**a**; white arrow). After surgery, computed tomography angiography demonstrated that the aneurysm was clipped (**b**). MCA = middle cerebral artery, ACA = anterior cerebral artery (Color figure online)

On admission, the patient was alert and neurologically intact but hypertensive and suffered from severe headache. While positioning for a head computed tomography (CT) scan, the patient became unresponsive and had sinus bradycardia (35 beats per minute). Pupils were still normal. Following intubation on the CT scan table, a head CT scan and a subsequent CT angiography (CTA) with an automated contrast injector system was performed. During CTA, systolic blood pressure raised suddenly over 200 mmHg and both pupils became non-reactive and dilated, despite sedation and antihypertensive medication. The CTA scan showed intracranial circulatory arrest at the level of proximal middle and anterior cerebral arteries (Fig. 1a, Supplementary Video 1). Extracranial arteries filled normally (Fig. 1a, Supplementary Video 1), confirming that CTA was performed properly. CT revealed diffuse subarachnoid hemorrhage (SAH) and a small intracerebral hematoma but no intracranial aneurysms (IAs). The patient was given a rapid infusion of mannitol, and the pupils normalized within 10 min. After stabilizing the patient, a follow-up CTA was performed 57 min after the first one. The scan showed a regained intracranial circulation (Fig. 1b, Supplementary Video 2), signs of a rebleed (Fig. 1b, Supplementary Video 2), and a suspicious lesion at the level of a right posterior communicating artery. In a following conventional catheter angiography one hour later, a right posterior communicating artery aneurysm was clearly visible (Fig. 2a). Based on a 12-lead electrocardiogram and biomarkers, no signs of stress cardiomyopathy (i.e., takotsubo cardiomyopathy) were observed during the hospitalization period.

#### Treatment and Outcome

Following angiography, the aneurysm was surgically clipped (Fig. 2b), and intracerebral and subdural hematomas were evacuated. Later, the patient developed hydrocephalus, which was treated with a ventriculoperitoneal shunt. The patient returned to work (higher education) one year after SAH, but due to neurocognitive sequelae, further rehabilitation was necessary. However, the patient was fully independent, lived at home, and continued sport activities, such as recreational running.

## Discussion

Sudden SAH death manifests as LOC and dilatation of pupils, and at least 40% of hospitalized SAH patients experience abnormal alteration of consciousness at the onset of SAH [3]. Fortunately, LOC is transient in many SAH patients [3]. Furthermore, a vast increase in the intracranial pressure (ICP) at the time of LOC is likely one of the major events that contribute to the beneficial formation of intra- and extraluminal thrombi, which aid to stop the bleeding from the ruptured aneurysm. In the presented case, the early CTA scan demonstrated the regained intracranial circulation (Fig. 1b, Supplementary Video 2) and a suspicious posterior communicating aneurysm, which most likely was partially thrombosed at the time of imaging.

Pathogenetic phenomena explaining transient LOC and pupillary dilatation in humans at the time of aneurysmal SAH have been speculated for a long time but never evidenced by CTA imaging. In the 1970s, Nornes and Magnaes investigated the dynamics of ICP in SAH patients during rebleedings and showed that an IA rupture may lead to ICP levels that exceed the diastolic blood pressure [4, 5]. Moreover, Nornes showed that circulation in the internal carotid artery stops at the end of diastole in aneurysmal SAH patients, most likely due to a rise in ICP [5]. These reports [4, 5] provided a better explanation for new hypotheses about intracranial events at the time of SAH. According to one widely accepted hypothesis, blood jetting through a ruptured aneurysm causes a rapid increase in ICP that in turn leads to pupillary dilatation. When ICP reaches the level of mean arterial pressure, cerebral perfusion pressure approaches zero, and intracranial circulatory arrest is presumed to happen. This is, however, the very first time that true but transient arrest of cerebral circulation has been documented.

In short, the presented images and clinical cascade document that at the time of aneurysm rupture, intracranial circulatory arrest happens simultaneously with pupillary dilatation and LOC. LOC increases the risk of poor outcome after SAH nearly threefold [3], but good recovery is evidently possible.

## Conclusion

Transient LOC in aneurysmal SAH patients is likely to be caused by a transient intracranial circulatory arrest, as has been speculated for a long time. In the described case, LOC did not accompany with transient cardiac arrest. Whether such simultaneous phenomenon occasionally happens remains to be documented.

## Electronic supplementary material

Below are the links to the electronic supplementary material.

**Supplementary Video 1**. Video shows axial reformats of a computed tomography angiography (CTA) scan at the time of aneurysm rupture and intracranial circulatory arrest. Anterior and middle cerebral arteries fill vaguely, whereas extracranial temporal arteries fill normally. (MOV 1958 kb)

**Supplementary Video 2**. Video shows axial reformats of a computed tomography angiography (CTA) scan after regaining intracranial circulation. (MOV 2302 kb)
